# Analyzing the Effect of Imputation on Classification Performance under MCAR and MAR Missing Mechanisms

**DOI:** 10.3390/e25030521

**Published:** 2023-03-17

**Authors:** Philip Buczak, Jian-Jia Chen, Markus Pauly

**Affiliations:** 1 Department of Statistics, TU Dortmund University, 44227 Dortmund, Germany; 2 Department of Computer Science, TU Dortmund University, 44227 Dortmund, Germany; 3 UA Ruhr, Research Center Trustworthy Data Science and Security, 44227 Dortmund, Germany

**Keywords:** missing values, imputation, MICE, missForest, classification, machine learning

## Abstract

Many datasets in statistical analyses contain missing values. As omitting observations containing missing entries may lead to information loss or greatly reduce the sample size, imputation is usually preferable. However, imputation can also introduce bias and impact the quality and validity of subsequent analysis. Focusing on binary classification problems, we analyzed how missing value imputation under MCAR as well as MAR missingness with different missing patterns affects the predictive performance of subsequent classification. To this end, we compared imputation methods such as several MICE variants, missForest, Hot Deck as well as mean imputation with regard to the classification performance achieved with commonly used classifiers such as Random Forest, Extreme Gradient Boosting, Support Vector Machine and regularized logistic regression. Our simulation results showed that Random Forest based imputation (i.e., MICE Random Forest and missForest) performed particularly well in most scenarios studied. In addition to these two methods, simple mean imputation also proved to be useful, especially when many features (covariates) contained missing values.

## 1. Introduction

Missing data is a reoccurring challenge in statistical analyses in the life sciences and many other domains from the information sciences. For example, patients may refuse to share sensitive details about their health. In repeated measurement designs, patients may either miss single measurements or drop out completely. Survey data may also be missing by design where not all respondents receive the same set of questions. Generally speaking, there are three different mechanisms to distinguish when handling missing data [1]. The *missing completely at random* (MCAR) mechanism assumes that missingness does not depend on the data (neither the observed nor the unobserved part). The other two mechanisms allow for missingness to depend on the data. For *missing at random* (MAR), the missingness depends on the observed components of an observation (i.e., the dependance relation is encoded in the observed data), while for *missing not at random* (MNAR), the missingness depends on the unobserved compontents of an observation (i.e., the dependance relation is not encoded in the observed data).

Thus, depending on the (usually unknown) missing mechanism that governs the occurrence of given missing values, the common practice of listwise deletion (i.e., deletion of observations that contain at least one missing value) may lead to reduced sample size and thus information loss. As a remedy, missing data is often imputed through different techniques such as simple mean or mode imputation, or advanced imputation methods such as MICE [2,3] or techniques stemming from machine learning (ML) [4,5,6,7]. However, data imputation may also affect the quality and validity of prediction or inference from resulting models [3,8,9,10,11]. It is therefore crucial to analyze the extent to which imputation methods influence subsequent regression or classification models obtained from imputed data. In the context of classification, a few studies have compared the performance of different imputation methods [12,13,14,15]. For our purposes most notably due to the variety of imputation and classification methods, Farfanghar et al. [12] compared the predictive performance of six different imputation methods w.r.t. the predictive performance of five classifiers. As imputation methods, the authors used Hot Deck imputation, Naive Bayes imputation, mean imputation as well as a polytomous regression-based imputation method. Additionally, the former two methods, were also embedded within a custom imputation framework meant to improve the performance of their standalone counterpart. Although they found that imputation generally improves performance, no imputation method was found to regularly outperform its competitors. Since their 2008 study, new imputation methods were suggested, for example, the Refs. [4,6]. In particular, tree-based ML approaches have shown some enhancements with respect to accuracy for regression problems [10,11,16]. For example, Ramosaj et al. [10] have recently analyzed how different imputation methods influence the subsequent predictive performance of linear regression and tree-based ML approaches. In their work, they found a certain preference to use the Random Forest based missForest [4] imputation method or a MICE [2] model based on Bayesian linear regression.

In light of their findings for the regression context, we investigated whether similar conclusions regarding the performance of the different imputation methods can be drawn for classification problems. Compared to the previous studies in the classification context, we used a more modern suite of imputation algorithms, that is, missForest and MICE, and further considered MCAR as well as MAR missing mechanisms with varying missing patterns. In the next section, we describe our simulation set-up in more detail. We report our results in Section 3 and follow up with a discussion in Section 4.

## 2. Materials and Methods

For our analysis, we used six binary classification problems from the life sciences. Table 1 provides an overview over the datasets including the number of observation and features as well as a short description of the target variable and features (covariates) used for prediction. The datasets *Phoneme* and *Pima Indians* were obtained from the Open Machine Learning Project [17], while the datasets *Haberman*, *Skin* [18], *SPECT* and *Wilt* [19] were obtained from the UCI Machine Learning Repository [20]. Except for the *Skin* dataset, we used the original datasets as they came. The Skin dataset had an original size of 245,057 observations. In our analysis, we used a random sample of 5% where the original class balance was preserved through stratified sampling. None of the original datasets included any missing values. To limit the scope of our investigation, we have decided to only focus on datasets with numerical features at this time.

**Figure 1:**
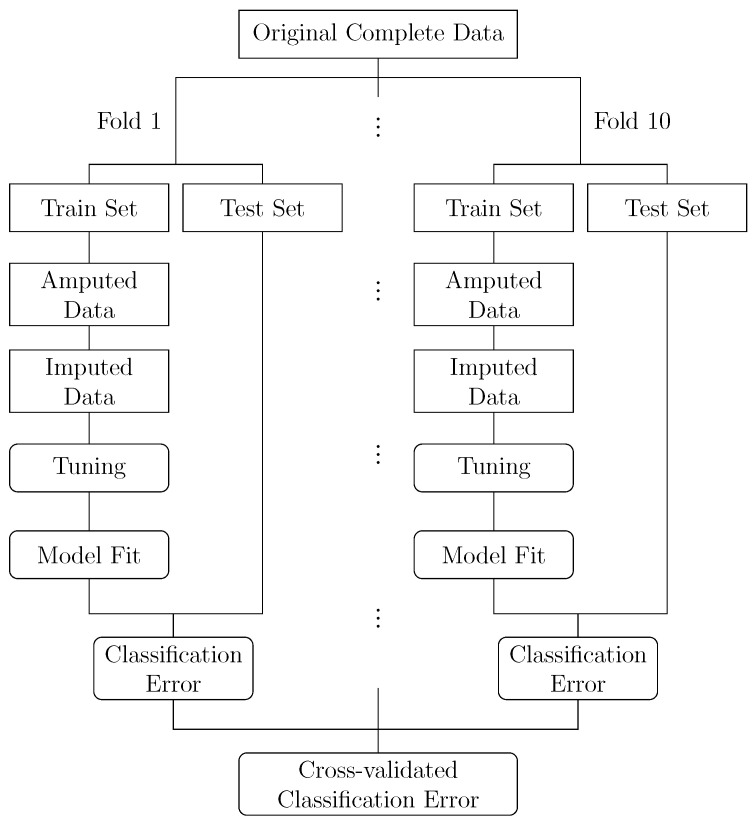
Workflow used in simulation study.

The general flow of our analysis is depicted in Figure 1. Starting from an original complete dataset, we generated ten train/test partitions as in a 10-fold cross-validation. In each fold, we generated missing values in the feature data of the respective training set. To this end, we used the *ampute* function from the MICE R package [2] which implements the multivariate amputation procedure proposed in [21]. We varied the proportion of missing values between 10%, 30% and 50%, that is, we set 10%, 30% and 50% of the original feature data as missing, respectively. The missingness was generated via a MCAR as well as different MAR mechanisms. One key component of the amputation procedure is the ability to specify a missingness pattern that governs which set of features may contain missing values and which set of features is kept complete. This allows for creating flexible missingness scenarios. To study different MAR mechanisms, we specified three missingness patterns that vary in the amount of features that may contain missing values and as such cover diverse scenarios. First, a pattern where for any given observation only one feature value at a time could be set missing. Second, a pattern where missing values could only occur in the middle third of the features. Third, a pattern where missing values could only occur in the first and last third of the features. We will refer to these three MAR patterns as the *One at a Time*, *One Third* and *Two Thirds* pattern, respectively.

Having introduced missingness into the training set, we then imputed the missing values using three MICE [3] algorithms as well as missForest [4], Hot Deck imputation [22] and a naive mean imputation. The three MICE algorithms we used were Bayesian linear regression (denoted as *MICE Norm*), Predictive Mean Matching (denoted as *MICE PMM*) and Random Forest (denoted as *MICE RF*). A common approach of the MICE algorithms and missForest is the concept of treating the imputation for a feature containing missing values as a prediction problem where the respective feature acts as the target variable that is predicted using the remaining features. Usually, some model (e.g., linear model or decision tree) is learned on the data subset for which the respective feature was observed. This idea is fleshed out in varying ways between the different imputation methods.

MICE Norm is based on Rubin’s [23] imputation method under the assumption of normality. The parameters of the linear model are sampled from their respective posterior distribution which is estimated using the observed data [3]. MICE PMM extends upon this by sampling a set of candidate donors (five per default) from the observed data whose values are closest to the predictions for missing data points as obtained from the Bayesian linear model. From this set of candidates, one donor is then chosen at random. Thus, PMM only imputes values that were actually observed and consequently does not suffer from the issue of out-of-range imputation [3]. For more details on the matching procedure see the Ref. [24].

MICE RF and missForest fall into the category of tree-based imputation methods. MICE RF is based on the algorithm proposed in Doove et al. [25] in which *k* individual tree models are fit on bootstrap samples from the observed data. The data point requiring imputation is then passed through each tree and falls into a terminal node, respectively. For each of these terminal nodes, one donor is sampled at random from all observations belonging to the node, thus resulting in a set of *k* donors overall. Out of this set, one donor is chosen at random for the imputation. One commonality of all MICE methods is the concept of multiple imputation. To account for the variability of the imputation process due to the probabilistic nature of the methods, multiple imputed datasets are created. Typically, subsequent analysis is performed on each dataset and the respective model results are pooled. Because we did not analyze uncertainty or perform inference, and in order to limit the computational complexity and to keep our simulation setup consistent, we aggregated the imputed datasets into a combined dataset. This was performed by averaging numeric features and selecting the mode for categorical features, respectively.

In contrast to MICE RF, missForest uses Random Forests to iteratively improve upon an initial imputation guess. The algorithm repeatedly cycles through all originally non-complete features and updates its imputations by fitting Random Forest models and obtaining new predictions for the missing entries. Since the features used in a respective step for prediction potentially contain imputed values themselves that were improved upon in previous steps, the procedure gradually refines its imputations over time. Another difference between MICE and missForest is that the latter does not use multiple imputation.

Last, Hot Deck imputation obtains imputations by sampling from the set of observed values where observations that are similar to the observation requiring imputation have a higher chance of being selected as the donor through proximity-based weighting. For imputing with Hot Deck imputation, MICE and missForest in R (version 4.0.0; [26]), we used the hot.deck package [27], the mice package [2] and the missRanger package [6], respectively. The latter allows for additional PMM and is a faster implementation of missForest since it uses the computationally efficient ranger package [28]. For MICE, we used the Bayesian linear regression (MICE Norm), Predictive Mean Matching (MICE PMM) and Random Forest (MICE RF) variants. For missForest, we included both a non-PMM and a PMM variant with three candidate non-missing values from which the imputed value was sampled. We used default values for MICE and missForest settings except for the number of trees and the maximum chaining iterations of missForest which we set to 100 and 3, respectively. The number of multiple imputations for MICE was five as per default. Having imputed the missing values, we continued with the task of classification. As classifiers, we used Elastic Net regularized logistic regression (denoted EN-LR in the following), Random Forest (RF), Support Vector Machine (SVM) and Extreme Gradient Boosting (XGBoost). All ML experiments were performed with the mlr package that provides a unified interface for ML-based analysis in R. For our classifiers, we used the glmnet package [29] for EN-LR, the ranger package [28] for RF, the e1071 package [30] for (radial basis) SVM and the xgboost package [31] for XGBoost. All of these learners have individual sets of hyperparameters that must be specified in advance. Their optimal choice is problem-dependent and approximated via hyperparameter tuning. Incorporating tuning into a benchmark experiment of different ML algorithms requires a nested resampling approach, where tuning is performed in the inner, and validation in the outer resampling loop. Otherwise, tuning and validating on overlapping data samples may lead to optimistic error estimates due to overfitting [32]. Thus, we perform an additional 3-fold cross-validation for hyperparameter tuning on the respective imputed training sets. Table A1 shows the respective hyperparameters and search spaces considered for tuning via a random search with 30 iterations. For hyperparameters that were not tuned, we used the default values.

After tuning, the classification models were learned on the entire imputed training set using the optimal hyperparameter settings, and validated on the test set. For each fold, this yielded a classification performance as measured by the Mean Misclassification Error (MMCE), that is, the proportion of wrongly classified instances in relation to all instances. Averaging over the fold-specific performances resulted in an overall cross-validated classification performance on which further comparisons are based. For each combination of dataset, imputation method and learner, we performed 100 replications.

## 3. Results

Table 2 shows the mean ranks based on the MMCE achieved by the respective classifiers for the 100 replications under a MCAR mechanism. For each row in the table, the best (i.e., lowest) mean rank is signified by bold font and a grey-colored cell. We have prepared similar tables for the standard deviation of the MMCE values for all scenarios in the Appendix (Table A2, Table A3, Table A4 and Table A5). As the observed variability is low and homogeneous between the imputation methods, we will only focus on the MMCE ranks from now on. It can be seen that the optimal imputation method varied for the different classifiers.

For RF, SVM and XGBoost, MICE RF performed best overall by leading to the lowest mean MMCE ranks in more scenarios than the other imputation methods. For SVM, MICE PMM and missForest PMM were close in performance to MICE RF. In contrast to the other classifiers, MICE RF did not perform as well in the case of EN-LR. Instead, MICE PMM and mean imputation performed slightly better than the other imputation methods. Except for XGBoost, where MICE RF slightly suffered from the increased proportion of missing values while mean imputation benefitted from it, the proportion of missing values did not noticeably affect the results for RF, SVM and EN-LR. Overall, Hot Deck, MICE Norm and missForest imputation were less competitive in the MCAR case.

When looking at the results for the *One at a Time* MAR pattern, Table 3 shows that some of the results from the MCAR case carried over. MICE RF performed well again for RF, SVM and XGBoost winning about a third to a half of the scenarios. For XGBoost, however, MICE PMM and missForest performed similary well. For EN-LR, the results were less clear-cut as well with MICE PMM, MICE Norm and mean imputation similarly competing for the best performance. Overall, Hot Deck and missForest PMM imputation were not as competitive for this missing pattern. MICE Norm was only competitive for classification with EN-LR and fell behind for the other classifiers.

When using the *One Third* pattern for MAR missingness, Table 4 shows that missForest clearly outperformed the other imputation methods for classification with RF, SVM and XGBoost. For these three classifiers, missForest was consistently optimal under almost all missingness proportions for the Phoneme, Skin and Wilt datasets. The results were more mixed for EN-LR where MICE Norm and MICE RF performed slightly better than the other imputation methods. In contrast to the MCAR and default MAR mechanism where Hot Deck imputation fell behind in almost all scenarios, it regularly achieved the lowest mean MMCE ranks on the Haberman dataset.

Finally, Table 5 displays the results for the MAR missing mechanism using the *Two Thirds* pattern. It can be seen that in most scenarios either missForest or mean imputation led to the lowest mean MMCE rank. For EN-LR, missForest and mean imputation performed similarly. When using RF, mean imputation was optimal for nearly all combinations of dataset and missingness proportion. The results for SVM and XGBoost were tied, with missForest and mean imputation winning about a third of all scenarios each. The results for EN-LR and XGBoost were sensitive to the missingness proportion. For both classifiers, mean imputation benefitted similarly from an increased proportion of missing values. Apart from missForest and mean imputation as well as MICE Norm for EN-LR, the remaining imputation methods were seldomly competetive.

## 4. Discussion

In this work, we studied the effect of imputation on the classification error under different missing mechanisms and missing proportions. To this end, we compared seven imputation methods, namely Hot Deck imputation, MICE Norm, MICE RF, MICE PMM, missForest, missForest PMM and mean imputation. As classifiers, we used EN-LR, RF, SVM and XGBoost. In our simulation study, we found that the optimal imputation method depended on the classifier, missing mechanism, as well as missingness pattern.

For a MCAR mechanism, we found that imputation via MICE RF worked best for RF, SVM and XGBoost. For EN-LR, the results were more mixed. Between the three MAR missing patterns (*One at a Time*, *One Third* and *Two Thirds*) we studied, the results for the *One at a Time* missing pattern resembled the MCAR results the most. Since for this pattern, only one feature value at a time could be missing for any given observation, the range of possible dependency structures that can arise are limited. Compared to the other two patterns, this situation is most similar to the MCAR mechanism where no dependency structure is present. Further, since the *One at a Time* pattern was allowed to vary w.r.t. to the features selected for containing the missing value, whereas the *One Third* and *Two Thirds* had a fixed set of features (i.e., the middle third, or the first and last third, respectively) where missing values could occur, the former pattern leads to more uncertainty. As such, the results for MCAR and *One at a Time* MAR are plausible, because MICE is designed to handle imputation uncertainty through multiple imputation. The missForest method, on the other hand, does not use multiple imputation and accordingly performed better in scenarios that included less uncertainty, that is, when using the *One Third* and *Two Thirds* patterns where it was optimal for many combinations of dataset, classifier and missing proportion.

Concerning practical insights, our study showed that RF-based imputation worked well under all MCAR and MAR missing mechanisms considered here. However, the optimality of MICE RF and missForest varied depending on the missing mechanism and pattern. Thus, this needs to be considered when using either. Even though the missing mechanism is generally unknown in practice, it is often feasible to form some assumptions based on the data-generating process. In most scenarios one will find that the underlying missingness seldomly follows a true MCAR mechanism. Potential patterns of missingness can also be gauged from exploratory data analysis by analyzing the presence and frequency of missing values. Alternatively, we found mean imputation to be a viable option when many features contained missing values. However, there might be a caveat to this finding. We did not specifically simulate the data and its distribution, so we did not explicitly examine cases in which the assumptions of mean imputation are violated or challenged. Our findings in this regard might have benefitted from studying classification as opposed to regression tasks. Future research should examine the impact of missing and mean imputation for heavily skewed features, for instance.

As for the MICE results, it should be noted that our approach of aggregating the imputed datasets did not make use of MICE‘s inherent advantage of controlling for the between-imputation variability by performing model analysis on the individual imputed datasets and subsequently averaging the resulting models. When performing inference or studying uncertainty, this step is detrimental as otherwise resulting standard errors are overconfident or Type I errors inflated, respectively. As we were only interested in studying classification errors, we have decided for the data aggregation to keep the simulation setup more consistent for all imputation methods and to limit the computational complexity (as each imputed dataset would have required a costly individual hyperparameter tuning step). However, as some of the MICE methods were not as competitive in our simulation study, future work should (if the computational resources permit) study whether the fitting and averaging of classification models on the individual imputed datasets leads to different results regarding the classification error.

Future work could also include listwise deletion as a benchmark method. We have refrained from using it here since the nested resampling approach resulted in small data subsets in the inner cross-validation folds and reducing the sample sizes even more through listwise deletion led to numerical issues with the logistic regression classifier on the smaller datasets from our simulation. Thus, we have decided to exclude this benchmark method for reasons of consistency.

Overall, our results indicated the importance of not only considering the missing mechanism when imputing, but also the pattern of missingness. The fact that the imputation methods were quite sensitive to the pattern choice, warrants further research in the future to investigate the effect of missingness patterns on imputation quality in more detail. This also includes studying more realistic missingness patterns. In our simulation study, the distinction between features that could contain missing values and those that could not was imposed by their order in the dataset (e.g., missings could only occur in the middle third of the features). In theory, this may occur in survey scenarios where a part of the questions is blocked from certain respondents (e.g., through filter questions). However, while helping to standardize the simulation process for all the different datasets, this design choice did not realistically reflect the common occurrence of relationships between features where the value of one feature regulates the probability of missingness for another feature. For example, in an online survey context, older people may have higher probabilities of missing answers or not completing their survey since they may be more challenged by technical aspects of the survey than younger participants. In another example, in-person measurements could be affected by the place of residence where respondents living far away might be more inclined to miss measurements due to the long travel time or due to insufficient public transportation options. For future work, one could design missingness patterns to better reflect such phenomena and thus make them more realistic. Instead of randomly selecting the features that may contain missing values, one could also study how imputation is affected when missingness is induced in “important” features (as measured by variable importance measures for example). Furthermore, to limit the scope of our analysis we only considered datasets with numerical features. It would be interesting to study whether imputation for categorical features yields different results. This may also impact the performance of Hot Deck imputation which is more suitable to categorical features. To conclude, our work showed that (i) using modern RF imputation methods such as MICE RF or missForest may be favorable in terms of subsequent classification accuracy and that (ii) basing the choice of imputation method on the context in which they are to be used, may lead to improved classification performance.

## Figures and Tables

**Table 1:** Descriptions, class distributions and feature counts of datasets used in simulation study.

Dataset	Class 1	Class 0	Features	Description
*Haberman*	225	81	3	Survival status of breast cancer patients using age at operation,
				year of operation and number of positive axillary lymph nodes
*Phoneme*	3818	1586	5	Classification of oral and nasal sounds using five frequency-
				related characteristics of the sound sample
*Pima Indians*	500	268	8	Diabetes status in indigenous population using features such as
				BMI, blood pressure, insulin level, etc.
*Skin*	2542	9709	3	Segmentation of skin texture based on random samples of
				RGB color values from face images
*SPECT*	95	254	44	Diagnosis of computed tomography using information about
				22 regions of interest in stress and rest mode
*Wilt*	4578	261	5	Detection of diseased trees in segments of pansharpened
				images using spectral and texture information

**Table 2:** Mean MMCE ranks (lower = better) for imputation methods under a MCAR mechanism. Best value per row printed in bold and colored grey.

Classifier	% Miss.	Dataset	Hot Deck	MICE Norm	MICE PMM	MICE RF	missForest	missForest PMM	Mean
EN-LR	10%	Haberman	4.16	4.02	4.08	3.83	**3.76**	4.32	3.85
		Phoneme	5.66	4.61	4.57	3.49	3.48	4.62	**1.57**
		Pima Indians	4.40	3.67	4.38	**3.38**	4.34	4.03	3.81
		Skin	4.19	1.59	**1.48**	2.97	5.01	5.99	6.77
		SPECT	4.44	3.92	3.87	**3.67**	3.96	4.06	4.08
		Wilt	4.47	3.43	**3.20**	3.39	4.08	4.66	4.76
	30%	Haberman	4.32	4.03	3.63	4.29	3.84	4.34	**3.56**
		Phoneme	6.60	4.46	4.76	3.01	3.25	4.86	**1.07**
		Pima Indians	4.20	**3.74**	**3.74**	4.12	4.10	4.18	3.91
		Skin	4.63	1.76	**1.24**	3.00	4.65	5.89	6.82
		SPECT	5.00	4.19	3.88	3.60	**3.40**	3.92	4.02
		Wilt	5.73	2.02	**1.97**	3.10	4.10	5.34	5.74
	50%	Haberman	4.54	3.80	3.84	3.93	3.75	4.41	**3.74**
		Phoneme	6.92	4.26	4.62	2.79	3.07	5.33	**1.01**
		Pima Indians	4.22	3.83	3.61	**3.55**	3.63	4.53	4.62
		Skin	5.05	1.70	**1.44**	2.89	3.99	5.93	7.00
		SPECT	5.24	3.80	3.90	3.71	3.88	**3.62**	3.87
		Wilt	5.50	1.64	**1.44**	3.01	5.43	5.50	5.50
RF	10%	Haberman	4.14	**3.81**	4.39	3.94	3.94	3.90	3.90
		Phoneme	4.43	4.31	3.92	**2.90**	3.75	4.00	4.69
		Pima Indians	3.98	3.94	4.15	**3.88**	4.11	4.03	3.92
		Skin	4.66	3.96	3.17	**1.36**	4.64	4.21	6.00
		SPECT	3.88	4.08	**3.71**	4.39	4.12	3.78	4.04
		Wilt	6.23	2.85	2.71	**2.62**	4.24	4.80	4.54
	30%	Haberman	4.34	3.93	3.96	4.41	3.92	3.72	**3.71**
		Phoneme	4.88	5.38	3.89	**2.10**	3.18	3.89	4.70
		Pima Indians	4.02	4.06	4.01	4.44	4.24	3.75	**3.49**
		Skin	5.46	4.50	3.12	**1.02**	6.18	5.21	2.50
		SPECT	4.15	3.82	4.14	4.09	4.10	**3.79**	3.92
		Wilt	6.85	**1.98**	2.10	2.67	5.20	5.80	3.41
	50%	Haberman	4.16	**3.62**	4.24	4.02	3.78	4.08	4.12
		Phoneme	5.57	5.48	4.06	**1.62**	2.71	4.36	4.20
		Pima Indians	4.24	4.16	4.19	3.98	3.91	3.84	**3.68**
		Skin	6.08	3.94	3.18	**1.04**	5.83	5.94	1.99
		SPECT	4.28	3.86	3.99	4.02	3.98	**3.76**	4.12
		Wilt	6.86	2.16	**1.61**	3.04	5.21	5.93	3.18
SVM	10%	Haberman	3.68	4.00	4.26	4.03	**3.64**	4.28	4.11
		Phoneme	4.04	4.70	3.79	**3.21**	3.90	3.90	4.45
		Pima Indians	**3.71**	**3.71**	3.92	4.32	4.22	3.89	4.22
		Skin	5.34	3.52	2.51	**1.32**	4.57	4.27	6.48
		SPECT	5.24	4.10	**3.31**	3.81	4.11	3.76	3.68
		Wilt	5.96	2.01	**1.74**	2.34	4.22	4.80	6.93
	30%	Haberman	4.07	4.28	4.12	4.16	4.06	**3.50**	3.79
		Phoneme	3.88	5.84	4.96	**1.91**	2.90	3.98	4.53
		Pima Indians	3.98	3.94	3.98	3.96	3.98	4.28	**3.88**
		Skin	5.66	3.30	2.22	**1.02**	5.33	5.11	5.36
		SPECT	6.56	3.61	**3.34**	3.44	3.61	3.81	3.64
		Wilt	6.01	2.24	**1.29**	2.46	4.20	4.82	6.98
	50%	Haberman	**3.80**	3.83	4.38	4.18	**3.80**	**3.80**	4.22
		Phoneme	4.31	5.94	5.26	**1.42**	2.86	4.41	3.80
		Pima Indians	3.94	4.36	4.04	3.80	4.07	**3.64**	4.16
		Skin	6.42	3.08	2.14	**1.00**	5.34	5.64	4.39
		SPECT	6.95	3.46	3.46	3.52	3.60	**3.41**	3.61
		Wilt	6.03	2.32	**1.09**	2.59	4.06	4.95	6.96
XGB	10%	Haberman	4.28	**3.79**	4.22	4.04	**3.79**	3.86	4.03
		Phoneme	4.26	4.34	4.37	**3.38**	3.51	4.06	4.08
		Pima Indians	3.95	4.25	3.98	**3.87**	4.04	4.04	3.88
		Skin	5.84	4.26	2.97	**1.47**	5.12	5.64	2.70
		SPECT	**3.77**	3.85	3.78	4.11	4.22	4.12	4.16
		Wilt	6.11	2.50	**2.26**	2.48	4.99	5.39	4.27
	30%	Haberman	4.28	3.90	3.93	4.07	4.10	4.17	**3.56**
		Phoneme	5.22	4.82	3.90	**2.41**	3.44	4.06	4.14
		Pima Indians	4.12	**3.64**	4.32	3.78	3.88	4.21	4.06
		Skin	6.10	4.02	3.09	**1.49**	5.79	5.79	1.73
		SPECT	3.88	3.96	4.08	4.22	3.94	4.27	**3.65**
		Wilt	6.72	**2.08**	2.11	2.40	5.19	5.89	3.61
	50%	Haberman	4.30	4.04	4.26	4.05	**3.50**	4.00	3.87
		Phoneme	5.76	4.78	4.00	**2.01**	3.22	4.48	3.76
		Pima Indians	4.11	3.87	3.84	4.40	3.88	4.24	**3.66**
		Skin	6.20	3.85	3.19	1.61	5.39	6.33	**1.43**
		SPECT	4.14	**3.73**	4.11	3.81	4.08	4.25	3.88
		Wilt	6.68	2.29	**1.92**	2.92	5.36	5.94	2.88

**Table 3:** Mean MMCE ranks (lower = better) for imputation methods under a MAR mechanism with *One at a Time* pattern. Best value per row printed in bold and colored grey.

Classifier	% Miss.	Dataset	Hot Deck	MICE Norm	MICE PMM	MICE RF	missForest	missForest PMM	Mean
EN-LR	10%	Haberman	4.52	**3.62**	4.16	3.65	3.94	4.19	3.92
		Phoneme	5.41	4.37	4.67	3.56	3.69	4.60	**1.70**
		Pima Indians	4.54	3.94	4.01	4.00	**3.41**	3.96	4.14
		Skin	4.61	1.65	**1.47**	2.90	5.11	5.48	6.79
		SPECT	4.43	3.82	3.83	**3.65**	4.12	4.14	4.01
		Wilt	4.30	3.64	**3.57**	3.73	3.68	3.74	5.34
	30%	Haberman	4.51	4.09	**3.64**	3.76	4.11	4.11	3.78
		Phoneme	6.55	4.15	4.48	3.29	3.46	5.01	**1.07**
		Pima Indians	4.09	**3.85**	4.04	4.02	3.94	3.97	4.09
		Skin	4.60	1.67	**1.33**	3.00	4.98	6.19	6.23
		SPECT	5.06	4.06	**3.60**	3.76	3.69	3.67	4.17
		Wilt	5.54	**1.74**	3.03	3.46	3.81	4.87	5.54
	50%	Haberman	4.78	3.70	3.69	4.28	3.78	4.20	**3.57**
		Phoneme	6.87	3.70	4.64	3.12	3.40	5.26	**1.01**
		Pima Indians	4.38	**3.48**	3.60	3.77	3.92	4.32	4.53
		Skin	5.04	1.66	**1.51**	2.83	4.37	5.61	6.98
		SPECT	5.54	3.85	3.60	3.87	3.69	**3.44**	4.00
		Wilt	5.42	**1.29**	1.92	3.38	5.15	5.42	5.42
RF	10%	Haberman	**3.48**	3.97	4.16	3.96	3.82	4.00	4.62
		Phoneme	4.36	4.37	4.09	**3.30**	3.52	4.10	4.26
		Pima Indians	4.21	4.24	4.21	**3.59**	3.96	3.79	4.00
		Skin	5.26	3.66	2.84	**1.42**	4.33	4.61	5.88
		SPECT	4.50	3.96	3.83	3.88	**3.60**	4.02	4.22
		Wilt	6.34	2.56	2.78	**2.46**	4.10	5.01	4.74
	30%	Haberman	4.54	3.67	4.06	4.02	**3.62**	4.09	4.00
		Phoneme	5.14	4.93	4.06	**1.90**	3.27	4.54	4.16
		Pima Indians	4.41	3.76	4.64	**3.67**	3.72	3.88	3.92
		Skin	6.17	3.52	2.82	**1.04**	5.73	5.99	2.73
		SPECT	3.96	4.02	3.90	4.25	**3.63**	4.03	4.22
		Wilt	6.89	2.14	**1.93**	2.55	4.95	5.94	3.60
	50%	Haberman	4.38	**3.56**	3.83	3.76	3.88	4.26	4.34
		Phoneme	5.60	5.15	3.78	**1.37**	3.04	4.95	4.12
		Pima Indians	4.32	3.85	4.08	3.90	3.93	**3.70**	4.20
		Skin	6.28	3.99	2.88	**1.01**	5.57	6.10	2.17
		SPECT	4.20	3.62	4.22	4.18	**3.60**	4.01	4.18
		Wilt	6.81	2.06	**1.84**	2.63	5.30	5.89	3.46
SVM	10%	Haberman	4.00	4.13	3.83	4.28	3.90	**3.76**	4.11
		Phoneme	3.88	4.38	4.20	**3.29**	3.30	3.87	5.10
		Pima Indians	4.42	3.90	4.28	**3.74**	3.86	3.99	3.83
		Skin	5.36	3.06	2.28	**1.64**	4.35	4.84	6.46
		SPECT	5.61	4.04	3.83	**3.47**	3.53	3.76	3.77
		Wilt	6.12	2.10	**1.93**	2.24	4.12	4.86	6.62
	30%	Haberman	3.72	3.90	4.20	4.43	4.08	3.94	**3.71**
		Phoneme	4.19	5.40	4.86	**1.68**	2.84	4.44	4.59
		Pima Indians	4.14	3.92	4.22	3.98	**3.83**	3.88	4.03
		Skin	6.12	2.84	2.19	**1.10**	5.22	5.70	4.82
		SPECT	6.75	3.38	**3.31**	3.82	3.86	3.44	3.46
		Wilt	6.16	2.14	**1.45**	2.41	4.12	4.94	6.78
	50%	Haberman	3.83	4.12	3.90	4.32	4.12	3.90	**3.82**
		Phoneme	5.00	5.58	4.53	**1.19**	2.76	4.99	3.95
		Pima Indians	**3.90**	3.92	4.14	4.00	4.04	3.92	4.09
		Skin	6.62	2.94	2.18	**1.01**	5.28	5.72	4.24
		SPECT	6.97	3.38	3.31	3.80	3.66	3.71	**3.17**
		Wilt	6.13	2.20	**1.25**	2.54	4.11	4.90	6.86
XGB	10%	Haberman	3.96	4.45	3.93	**3.71**	4.10	3.85	4.00
		Phoneme	4.24	3.85	4.18	3.48	**3.46**	4.05	4.74
		Pima Indians	4.04	**3.80**	4.11	3.83	4.01	3.98	4.24
		Skin	5.87	3.29	2.74	**1.60**	5.03	5.86	3.60
		SPECT	4.23	4.16	3.83	3.90	**3.77**	3.98	4.14
		Wilt	6.18	2.42	**2.26**	2.75	4.59	5.28	4.54
	30%	Haberman	4.43	**3.50**	4.24	3.87	3.78	4.27	3.90
		Phoneme	5.59	4.34	3.88	**2.35**	3.59	4.53	3.73
		Pima Indians	3.96	4.10	3.88	3.90	3.86	**3.83**	4.47
		Skin	6.42	3.59	2.77	**1.40**	5.45	6.09	2.29
		SPECT	3.74	4.16	4.16	4.27	**3.69**	4.00	3.97
		Wilt	6.74	2.10	**2.00**	2.37	4.99	5.84	3.96
	50%	Haberman	4.55	3.80	3.67	**3.57**	4.04	4.79	3.58
		Phoneme	5.79	4.87	4.02	**1.85**	3.39	4.74	3.33
		Pima Indians	4.14	3.95	**3.52**	4.08	4.14	3.93	4.24
		Skin	6.51	3.85	2.93	**1.44**	5.39	6.09	1.79
		SPECT	3.98	3.89	4.11	3.82	**3.80**	4.35	4.06
		Wilt	6.61	1.99	**1.90**	2.61	5.33	6.02	3.56

**Table 4:** Mean MMCE ranks (lower = better) for imputation methods under a MAR mechanism with *One Third* pattern. Best value per row printed in bold and colored grey.

Classifier	% Miss.	Dataset	Hot Deck	MICE Norm	MICE PMM	MICE RF	missForest	missForest PMM	Mean
EN-LR	10%	Haberman	3.78	4.43	3.85	4.10	**3.73**	4.24	3.86
		Phoneme	4.94	2.40	3.90	4.78	4.94	5.10	**1.95**
		Pima Indians	4.43	3.79	4.00	4.36	3.88	3.96	**3.58**
		Skin	5.97	**1.31**	3.57	4.36	3.22	2.58	7.00
		SPECT	4.78	4.43	3.94	3.70	**3.13**	3.92	4.10
		Wilt	5.42	3.40	3.27	**2.76**	3.85	5.40	3.90
	30%	Haberman	**3.64**	4.09	4.17	3.98	3.96	4.08	4.08
		Phoneme	6.57	3.99	6.17	2.68	3.85	3.15	**1.59**
		Pima Indians	3.99	3.92	4.14	**3.81**	4.22	3.93	3.98
		Skin	6.00	**1.29**	4.12	4.64	2.76	2.19	7.00
		SPECT	5.68	4.24	4.02	3.60	**2.86**	3.46	4.14
		Wilt	6.70	**1.65**	1.78	4.19	2.64	4.84	6.20
	50%	Haberman	**3.72**	3.94	4.01	4.30	3.96	3.73	4.34
		Phoneme	7.00	4.52	5.40	**1.90**	3.85	1.98	3.36
		Pima Indians	4.35	**3.67**	4.04	3.81	4.04	4.09	4.01
		Skin	6.00	2.00	4.18	4.66	2.18	**1.97**	7.00
		SPECT	4.58	3.92	3.73	**3.71**	3.84	3.78	4.44
		Wilt	6.34	**1.46**	1.61	4.03	2.92	4.99	6.64
RF	10%	Haberman	3.86	3.94	4.26	4.26	4.10	**3.36**	4.22
		Phoneme	5.53	4.32	4.64	3.60	**2.86**	3.78	3.27
		Pima Indians	3.88	**3.58**	3.68	4.20	4.12	3.95	4.58
		Skin	7.00	3.89	4.11	2.57	**1.58**	3.46	5.39
		SPECT	4.17	3.87	4.02	4.24	3.79	4.20	**3.71**
		Wilt	6.95	3.34	2.72	3.08	**2.21**	4.15	5.55
	30%	Haberman	**2.85**	4.20	4.16	4.28	4.23	4.00	4.30
		Phoneme	6.46	5.27	5.04	2.89	**1.99**	3.27	3.07
		Pima Indians	3.98	4.01	**3.56**	4.16	4.24	3.83	4.22
		Skin	7.00	4.92	4.28	2.27	**1.05**	4.77	3.71
		SPECT	5.24	4.11	3.39	3.60	4.58	4.14	**2.94**
		Wilt	7.00	2.69	2.15	4.06	**1.85**	5.98	4.26
	50%	Haberman	**3.12**	4.39	4.14	4.22	4.02	3.42	4.70
		Phoneme	6.54	5.18	5.36	3.06	**1.91**	3.77	2.19
		Pima Indians	3.83	3.69	3.77	4.19	4.34	**3.50**	4.68
		Skin	7.00	4.76	4.42	2.94	**1.09**	5.62	2.16
		SPECT	4.86	3.51	3.80	3.81	5.30	4.84	**1.89**
		Wilt	7.00	2.66	2.65	4.88	**1.45**	6.00	3.36
SVM	10%	Haberman	3.97	4.18	4.18	**3.69**	3.81	3.79	4.38
		Phoneme	5.00	4.38	4.94	3.54	**2.87**	3.51	3.76
		Pima Indians	**3.72**	4.28	4.16	3.82	3.96	4.05	4.01
		Skin	6.99	3.88	3.90	3.29	**1.66**	3.73	4.55
		SPECT	4.85	3.96	**3.64**	3.83	3.70	3.83	4.18
		Wilt	6.22	2.94	2.44	2.87	**2.06**	4.69	6.78
	30%	Haberman	**3.52**	4.29	4.28	3.77	3.97	3.77	4.40
		Phoneme	5.61	5.63	5.85	2.65	**2.01**	2.83	3.42
		Pima Indians	**3.56**	4.24	4.03	4.58	3.98	3.63	3.98
		Skin	7.00	4.38	4.75	2.90	**1.16**	4.84	2.98
		SPECT	5.64	3.51	**3.16**	3.47	4.22	3.37	4.62
		Wilt	7.00	3.10	2.18	3.62	**1.11**	5.00	6.00
	50%	Haberman	**3.29**	4.60	4.50	3.66	4.20	3.61	4.14
		Phoneme	5.74	5.62	5.88	2.54	**1.61**	2.64	3.97
		Pima Indians	3.29	4.12	3.97	3.92	4.92	**2.91**	4.86
		Skin	7.00	4.62	4.70	3.12	**1.28**	5.36	1.92
		SPECT	4.97	3.18	3.36	3.54	4.86	**2.00**	6.10
		Wilt	7.00	2.80	2.38	3.82	**1.01**	5.05	5.95
XGB	10%	Haberman	**3.66**	4.16	3.94	4.18	4.13	3.88	4.05
		Phoneme	4.70	4.20	4.72	3.62	**3.10**	3.79	3.87
		Pima Indians	4.12	3.80	4.30	3.94	**3.68**	4.22	3.92
		Skin	6.86	3.27	3.27	2.64	**2.07**	4.28	5.62
		SPECT	3.67	4.08	4.36	4.49	4.21	**3.59**	**3.59**
		Wilt	6.97	2.73	**2.47**	3.63	2.73	4.94	4.52
	30%	Haberman	**3.82**	4.14	4.30	**3.82**	3.96	3.95	4.03
		Phoneme	5.47	5.41	5.34	3.20	**2.22**	3.25	3.10
		Pima Indians	**3.50**	4.18	3.94	4.20	4.26	3.97	3.96
		Skin	6.98	3.97	4.27	2.44	**1.46**	5.51	3.38
		SPECT	3.41	4.30	4.04	4.53	5.34	4.06	**2.33**
		Wilt	7.00	2.56	2.54	4.00	**2.47**	5.97	3.44
	50%	Haberman	3.65	4.30	4.40	4.27	3.66	**3.60**	4.12
		Phoneme	5.82	5.53	5.51	3.31	**1.91**	3.53	2.39
		Pima Indians	3.74	4.04	4.02	3.79	4.27	**3.64**	4.50
		Skin	6.99	4.29	4.53	3.08	**1.29**	5.86	1.97
		SPECT	3.04	4.12	4.33	4.55	6.16	3.60	**2.20**
		Wilt	7.00	2.73	2.71	4.86	**1.73**	6.00	2.98

**Table 5:** Mean MMCE ranks (lower = better) for imputation methods under a MAR mechanism with *Two Thirds* pattern. Best value per row printed in bold and colored grey.

Classifier	% Miss.	Dataset	Hot Deck	MICE Norm	MICE PMM	MICE RF	missForest	missForest PMM	Mean
EN-LR	10%	Haberman	4.48	3.92	3.85	3.81	**3.72**	4.41	3.81
		Phoneme	6.81	5.36	4.32	2.99	1.77	5.49	**1.26**
		Pima Indians	4.46	3.61	3.87	4.57	**3.19**	3.72	4.57
		Skin	7.00	2.46	**1.76**	4.02	1.99	6.00	4.78
		SPECT	**3.83**	4.18	3.94	4.14	3.92	4.03	3.96
		Wilt	4.32	**2.13**	4.34	4.33	4.14	4.43	4.32
	30%	Haberman	4.90	4.14	3.82	3.41	**3.37**	4.96	3.41
		Phoneme	5.62	6.42	5.86	2.99	1.93	4.10	**1.08**
		Pima Indians	5.77	3.94	3.58	3.62	**3.54**	3.81	3.74
		Skin	6.99	**1.00**	3.66	4.56	3.46	6.01	2.32
		SPECT	5.38	4.82	4.22	**2.96**	3.15	3.19	4.28
		Wilt	4.38	**1.00**	4.57	4.62	4.57	4.47	4.38
	50%	Haberman	4.72	4.02	4.11	4.07	3.42	4.72	**2.94**
		Phoneme	5.82	5.96	5.38	2.97	**1.14**	4.82	1.92
		Pima Indians	6.74	3.46	3.70	2.87	3.00	5.50	**2.72**
		Skin	7.00	2.06	3.24	4.89	3.81	6.00	**1.00**
		SPECT	5.69	5.11	4.32	2.85	**1.97**	3.16	4.90
		Wilt	4.47	**1.00**	4.47	4.47	4.62	4.50	4.47
RF	10%	Haberman	4.30	**3.40**	3.81	3.94	4.21	4.23	4.12
		Phoneme	5.43	3.84	3.44	4.02	3.40	4.64	**3.23**
		Pima Indians	4.01	4.01	3.76	3.98	3.98	4.87	**3.40**
		Skin	3.90	3.68	5.53	4.68	3.63	4.80	**1.78**
		SPECT	4.23	4.34	4.14	3.71	3.69	**3.56**	4.32
		Wilt	7.00	3.36	2.54	3.39	**2.19**	4.36	5.16
	30%	Haberman	4.70	**2.98**	3.66	4.02	4.26	4.40	3.97
		Phoneme	6.48	3.94	3.66	3.59	2.53	6.18	**1.62**
		Pima Indians	4.88	3.83	3.95	3.59	3.36	5.52	**2.88**
		Skin	6.27	3.60	4.76	3.64	1.81	6.68	**1.24**
		SPECT	4.54	4.24	3.64	4.53	3.81	4.34	**2.90**
		Wilt	7.00	2.48	**1.89**	4.07	2.16	5.98	4.42
	50%	Haberman	4.72	**3.10**	3.90	3.98	3.72	4.74	3.84
		Phoneme	6.32	3.99	3.84	3.89	2.17	6.66	**1.13**
		Pima Indians	5.85	3.71	3.67	3.18	2.56	6.50	**2.52**
		Skin	6.93	4.53	3.68	3.79	1.88	6.07	**1.12**
		SPECT	4.78	4.46	4.03	4.00	4.16	5.24	**1.32**
		Wilt	7.00	2.08	**1.71**	4.82	2.44	6.00	3.94
SVM	10%	Haberman	**3.53**	3.90	4.34	4.04	3.85	4.17	4.18
		Phoneme	4.78	4.26	4.28	4.24	3.47	5.26	**1.72**
		Pima Indians	4.28	3.87	**3.63**	4.18	3.94	3.92	4.18
		Skin	3.85	3.83	5.19	5.00	5.31	3.44	**1.39**
		SPECT	4.76	4.23	3.92	3.98	**2.95**	3.81	4.36
		Wilt	7.00	2.65	**2.29**	2.77	2.50	4.86	5.93
	30%	Haberman	4.08	4.30	4.14	3.81	**3.76**	3.85	4.08
		Phoneme	5.78	4.39	3.97	3.94	2.25	6.63	**1.04**
		Pima Indians	4.40	4.04	3.78	3.65	**3.58**	4.64	3.90
		Skin	4.92	2.45	3.55	3.03	6.38	6.62	**1.04**
		SPECT	3.79	4.52	4.08	4.79	**2.69**	3.95	4.18
		Wilt	7.00	2.89	**1.86**	3.38	1.89	5.70	5.28
	50%	Haberman	3.86	4.06	4.14	4.14	**3.54**	4.15	4.11
		Phoneme	6.15	3.71	3.74	4.35	2.20	6.85	**1.00**
		Pima Indians	5.99	3.69	3.56	2.78	**2.44**	6.55	2.99
		Skin	5.07	2.48	3.08	3.42	5.93	7.00	**1.03**
		SPECT	**2.22**	4.33	4.44	4.74	3.77	3.62	4.88
		Wilt	7.00	2.71	**1.60**	4.18	1.69	6.00	4.82
XGB	10%	Haberman	4.42	**3.71**	3.75	4.14	3.94	4.31	3.72
		Phoneme	5.55	3.96	3.50	3.59	**2.88**	4.89	3.63
		Pima Indians	3.83	3.88	3.76	3.85	**3.55**	4.57	4.57
		Skin	6.50	4.08	4.54	3.93	**1.72**	5.18	2.06
		SPECT	3.90	4.24	4.30	3.86	4.28	3.86	**3.58**
		Wilt	6.99	3.02	3.23	**2.85**	2.88	4.78	4.24
	30%	Haberman	4.47	**3.52**	3.76	3.54	4.01	4.98	3.72
		Phoneme	6.27	3.80	3.49	3.55	**1.92**	6.68	2.29
		Pima Indians	4.40	4.28	4.13	3.60	**2.94**	5.24	3.39
		Skin	6.26	3.66	4.53	3.69	**1.49**	6.74	1.62
		SPECT	3.67	4.87	4.50	4.58	4.40	4.18	**1.80**
		Wilt	7.00	2.56	**2.45**	3.69	2.66	5.96	3.68
	50%	Haberman	4.67	**3.27**	4.00	3.87	3.78	4.59	3.82
		Phoneme	6.09	3.88	3.56	4.20	2.13	6.91	**1.22**
		Pima Indians	5.40	4.03	3.86	3.29	**2.56**	6.08	2.80
		Skin	6.59	4.39	3.91	3.66	1.58	6.41	**1.46**
		SPECT	3.67	5.24	4.76	4.44	4.72	4.12	**1.04**
		Wilt	7.00	2.43	**2.29**	4.81	2.93	6.00	2.54

## Data Availability

The *Phoneme* and *Pima Indians* datasets were obtained from the Open Machine Learning Project [17]. The *Haberman*, *Skin*, *SPECT* and *Wilt* datasets were obtained from the UCI Machine Learning Repository [20]. The R script for our simulation study is available at our OSF Repository https://osf.io/3z9sb/ (accessed on 10 March 2023).

## Appendix A

**Table A1:** Hyperparameters and search spaces for tuning.

Classifier	Hyperparameter	Search Space	Transformation
EN-LR	alpha	[0,1]	–
	lambda	[−15,15]	$2^{x}$
RF	mtry	{2,…,#features}	–
	min.node.size	{1,…,10}	–
	splitrule	{gini, extratrees}	–
SVM	cost	[−5,5]	$2^{x}$
	gamma	[−5,5]	$2^{x}$
XGBoost	nrounds	{10,…,200}	–
	max_depth	{1,…,20}	–
	eta	[0.1,0.5]	–
	lambda	[−1,0]	$10^{x}$

**Table A2:** Standard deviations of MMCE values for imputation methods under a MCAR mechanism.

Classifier	% Miss.	Dataset	Hot Deck	MICE Norm	MICE PMM	MICE RF	missForest	missForest PMM	Mean
EN-LR	10%	Haberman	0.007	0.008	0.008	0.008	0.007	0.008	0.007
		Phoneme	0.001	0.001	0.001	0.001	0.001	0.001	0.001
		Pima Indians	0.004	0.004	0.004	0.005	0.005	0.005	0.004
		Skin	0.000	0.000	0.000	0.000	0.001	0.000	0.000
		SPECT	0.013	0.011	0.012	0.011	0.011	0.011	0.011
		Wilt	0.034	0.036	0.030	0.036	0.031	0.040	0.028
	30%	Haberman	0.006	0.008	0.008	0.007	0.007	0.007	0.007
		Phoneme	0.001	0.001	0.001	0.001	0.001	0.001	0.001
		Pima Indians	0.004	0.004	0.005	0.005	0.005	0.004	0.005
		Skin	0.000	0.000	0.000	0.000	0.002	0.001	0.000
		SPECT	0.012	0.012	0.010	0.011	0.010	0.012	0.011
		Wilt	0.000	0.019	0.014	0.019	0.007	0.000	0.000
	50%	Haberman	0.007	0.008	0.007	0.008	0.007	0.007	0.008
		Phoneme	0.001	0.001	0.001	0.001	0.001	0.001	0.001
		Pima Indians	0.005	0.005	0.004	0.004	0.005	0.005	0.004
		Skin	0.001	0.000	0.000	0.000	0.002	0.002	0.000
		SPECT	0.014	0.011	0.011	0.011	0.011	0.011	0.012
		Wilt	0.000	0.001	0.001	0.009	0.000	0.000	0.000
RF	10%	Haberman	0.011	0.011	0.011	0.012	0.010	0.012	0.011
		Phoneme	0.002	0.002	0.002	0.002	0.002	0.002	0.002
		Pima Indians	0.007	0.007	0.006	0.007	0.007	0.007	0.006
		Skin	0.000	0.000	0.000	0.000	0.000	0.000	0.000
		SPECT	0.011	0.011	0.010	0.012	0.013	0.012	0.011
		Wilt	0.001	0.001	0.001	0.001	0.001	0.001	0.001
	30%	Haberman	0.016	0.011	0.012	0.014	0.012	0.013	0.012
		Phoneme	0.002	0.002	0.002	0.003	0.002	0.002	0.002
		Pima Indians	0.006	0.006	0.007	0.006	0.007	0.007	0.007
		Skin	0.000	0.000	0.000	0.000	0.000	0.000	0.000
		SPECT	0.011	0.011	0.011	0.012	0.012	0.012	0.011
		Wilt	0.001	0.001	0.001	0.001	0.001	0.001	0.001
	50%	Haberman	0.015	0.014	0.015	0.014	0.014	0.013	0.013
		Phoneme	0.003	0.003	0.002	0.002	0.002	0.003	0.002
		Pima Indians	0.008	0.007	0.006	0.006	0.007	0.008	0.007
		Skin	0.000	0.000	0.000	0.000	0.001	0.001	0.000
		SPECT	0.012	0.011	0.011	0.012	0.011	0.012	0.011
		Wilt	0.001	0.001	0.001	0.001	0.001	0.001	0.001
SVM	10%	Haberman	0.010	0.009	0.008	0.009	0.009	0.009	0.009
		Phoneme	0.003	0.003	0.003	0.003	0.003	0.003	0.002
		Pima Indians	0.006	0.006	0.006	0.006	0.006	0.006	0.007
		Skin	0.000	0.000	0.000	0.000	0.000	0.000	0.000
		SPECT	0.012	0.015	0.012	0.011	0.012	0.014	0.013
		Wilt	0.001	0.001	0.001	0.001	0.001	0.001	0.001
	30%	Haberman	0.010	0.010	0.009	0.008	0.011	0.008	0.009
		Phoneme	0.003	0.003	0.003	0.003	0.003	0.003	0.004
		Pima Indians	0.006	0.006	0.007	0.006	0.007	0.006	0.006
		Skin	0.000	0.000	0.000	0.000	0.000	0.000	0.000
		SPECT	0.013	0.013	0.013	0.012	0.013	0.013	0.013
		Wilt	0.001	0.001	0.001	0.001	0.001	0.001	0.001
	50%	Haberman	0.008	0.009	0.008	0.010	0.009	0.008	0.009
		Phoneme	0.003	0.003	0.003	0.003	0.003	0.003	0.003
		Pima Indians	0.007	0.007	0.006	0.006	0.006	0.007	0.007
		Skin	0.000	0.000	0.000	0.000	0.001	0.001	0.000
		SPECT	0.014	0.013	0.013	0.013	0.014	0.014	0.013
		Wilt	0.001	0.001	0.001	0.001	0.002	0.001	0.001
XGB	10%	Haberman	0.015	0.014	0.013	0.015	0.015	0.013	0.015
		Phoneme	0.002	0.003	0.002	0.003	0.002	0.002	0.002
		Pima Indians	0.009	0.010	0.008	0.008	0.009	0.009	0.009
		Skin	0.000	0.000	0.000	0.000	0.000	0.000	0.000
		SPECT	0.012	0.012	0.012	0.012	0.012	0.012	0.012
		Wilt	0.001	0.001	0.001	0.001	0.001	0.001	0.001
	30%	Haberman	0.015	0.015	0.015	0.016	0.015	0.015	0.014
		Phoneme	0.003	0.003	0.003	0.003	0.003	0.003	0.003
		Pima Indians	0.009	0.009	0.009	0.008	0.008	0.008	0.008
		Skin	0.001	0.000	0.000	0.000	0.001	0.001	0.000
		SPECT	0.012	0.011	0.012	0.013	0.012	0.011	0.012
		Wilt	0.001	0.001	0.001	0.001	0.001	0.001	0.001
	50%	Haberman	0.016	0.015	0.015	0.015	0.015	0.016	0.016
		Phoneme	0.003	0.003	0.003	0.003	0.003	0.003	0.003
		Pima Indians	0.008	0.009	0.009	0.009	0.010	0.009	0.008
		Skin	0.001	0.001	0.001	0.000	0.001	0.001	0.000
		SPECT	0.012	0.012	0.013	0.013	0.011	0.011	0.012
		Wilt	0.001	0.001	0.001	0.001	0.001	0.002	0.001

**Table A3:** Standard deviations of MMCE values for imputation methods under a MAR mechanism with *One at a Time* pattern.

Classifier	% Miss.	Dataset	Hot Deck	MICE Norm	MICE PMM	MICE RF	missForest	missForest PMM	Mean
EN-LR	10%	Haberman	0.007	0.007	0.007	0.008	0.008	0.008	0.007
		Phoneme	0.001	0.001	0.001	0.001	0.001	0.001	0.001
		Pima Indians	0.005	0.004	0.004	0.004	0.004	0.005	0.004
		Skin	0.000	0.000	0.000	0.000	0.001	0.001	0.000
		SPECT	0.012	0.011	0.012	0.010	0.011	0.011	0.011
		Wilt	0.014	0.041	0.036	0.033	0.016	0.021	0.000
	30%	Haberman	0.007	0.008	0.008	0.008	0.008	0.007	0.007
		Phoneme	0.001	0.001	0.001	0.001	0.001	0.001	0.001
		Pima Indians	0.005	0.004	0.005	0.004	0.004	0.005	0.005
		Skin	0.000	0.000	0.000	0.000	0.002	0.002	0.000
		SPECT	0.012	0.010	0.012	0.012	0.011	0.012	0.012
		Wilt	0.000	0.015	0.037	0.026	0.016	0.001	0.000
	50%	Haberman	0.007	0.008	0.008	0.007	0.008	0.006	0.007
		Phoneme	0.001	0.001	0.001	0.001	0.001	0.001	0.001
		Pima Indians	0.005	0.004	0.004	0.005	0.005	0.004	0.005
		Skin	0.001	0.000	0.000	0.000	0.003	0.002	0.001
		SPECT	0.013	0.010	0.012	0.011	0.011	0.011	0.011
		Wilt	0.000	0.006	0.010	0.016	0.006	0.000	0.000
RF	10%	Haberman	0.010	0.012	0.012	0.012	0.012	0.011	0.013
		Phoneme	0.002	0.002	0.002	0.002	0.002	0.002	0.002
		Pima Indians	0.006	0.006	0.006	0.007	0.006	0.006	0.006
		Skin	0.000	0.000	0.000	0.000	0.000	0.000	0.000
		SPECT	0.012	0.011	0.012	0.012	0.011	0.011	0.011
		Wilt	0.001	0.001	0.001	0.001	0.001	0.001	0.001
	30%	Haberman	0.013	0.013	0.011	0.013	0.014	0.014	0.014
		Phoneme	0.002	0.002	0.002	0.002	0.002	0.003	0.003
		Pima Indians	0.006	0.007	0.007	0.007	0.007	0.007	0.006
		Skin	0.000	0.000	0.000	0.000	0.000	0.000	0.000
		SPECT	0.011	0.011	0.010	0.012	0.011	0.011	0.011
		Wilt	0.001	0.001	0.001	0.001	0.001	0.001	0.001
	50%	Haberman	0.015	0.014	0.015	0.015	0.013	0.014	0.014
		Phoneme	0.003	0.003	0.003	0.002	0.003	0.002	0.002
		Pima Indians	0.007	0.007	0.007	0.007	0.007	0.007	0.007
		Skin	0.001	0.000	0.000	0.000	0.001	0.001	0.000
		SPECT	0.012	0.011	0.011	0.011	0.011	0.011	0.012
		Wilt	0.001	0.001	0.001	0.001	0.001	0.001	0.001
SVM	10%	Haberman	0.010	0.010	0.010	0.010	0.011	0.009	0.009
		Phoneme	0.003	0.003	0.003	0.002	0.003	0.003	0.003
		Pima Indians	0.006	0.005	0.007	0.007	0.006	0.006	0.006
		Skin	0.000	0.000	0.000	0.000	0.000	0.000	0.000
		SPECT	0.014	0.013	0.013	0.012	0.013	0.014	0.014
		Wilt	0.001	0.001	0.001	0.001	0.001	0.001	0.001
	30%	Haberman	0.009	0.011	0.009	0.009	0.010	0.011	0.009
		Phoneme	0.003	0.004	0.004	0.003	0.003	0.003	0.004
		Pima Indians	0.006	0.007	0.006	0.006	0.007	0.007	0.006
		Skin	0.000	0.000	0.000	0.000	0.001	0.001	0.000
		SPECT	0.013	0.012	0.013	0.013	0.013	0.013	0.012
		Wilt	0.001	0.001	0.001	0.001	0.001	0.001	0.001
	50%	Haberman	0.009	0.010	0.008	0.011	0.008	0.009	0.008
		Phoneme	0.003	0.003	0.003	0.003	0.004	0.003	0.003
		Pima Indians	0.007	0.007	0.007	0.007	0.007	0.007	0.007
		Skin	0.001	0.000	0.000	0.000	0.001	0.001	0.000
		SPECT	0.016	0.013	0.014	0.013	0.012	0.013	0.014
		Wilt	0.001	0.001	0.001	0.001	0.002	0.001	0.001
XGB	10%	Haberman	0.013	0.013	0.014	0.015	0.014	0.016	0.013
		Phoneme	0.002	0.002	0.002	0.002	0.003	0.002	0.003
		Pima Indians	0.008	0.009	0.008	0.008	0.009	0.010	0.009
		Skin	0.000	0.000	0.000	0.000	0.000	0.000	0.000
		SPECT	0.013	0.013	0.012	0.012	0.012	0.013	0.012
		Wilt	0.001	0.001	0.001	0.001	0.001	0.001	0.001
	30%	Haberman	0.014	0.013	0.014	0.016	0.015	0.014	0.015
		Phoneme	0.003	0.003	0.003	0.003	0.003	0.003	0.003
		Pima Indians	0.010	0.009	0.009	0.008	0.008	0.010	0.008
		Skin	0.000	0.000	0.000	0.000	0.001	0.001	0.000
		SPECT	0.013	0.013	0.013	0.014	0.013	0.013	0.012
		Wilt	0.001	0.001	0.001	0.001	0.001	0.001	0.001
	50%	Haberman	0.016	0.015	0.015	0.016	0.016	0.013	0.014
		Phoneme	0.003	0.003	0.003	0.003	0.003	0.003	0.002
		Pima Indians	0.009	0.010	0.010	0.009	0.009	0.009	0.008
		Skin	0.001	0.000	0.000	0.000	0.001	0.001	0.000
		SPECT	0.013	0.012	0.012	0.013	0.013	0.012	0.012
		Wilt	0.001	0.001	0.001	0.001	0.001	0.001	0.001

**Table A4:** Standard deviations of MMCE values for imputation methods under a MAR mechanism with *One Third* pattern.

Classifier	% Miss.	Dataset	Hot Deck	MICE Norm	MICE PMM	MICE RF	missForest	missForest PMM	Mean
EN-LR	10%	Haberman	0.007	0.007	0.007	0.007	0.007	0.007	0.008
		Phoneme	0.001	0.001	0.001	0.001	0.001	0.001	0.001
		Pima Indians	0.004	0.005	0.005	0.005	0.005	0.005	0.004
		Skin	0.000	0.000	0.000	0.000	0.000	0.000	0.000
		SPECT	0.011	0.011	0.012	0.013	0.012	0.012	0.012
		Wilt	0.001	0.029	0.033	0.019	0.037	0.037	0.001
	30%	Haberman	0.008	0.007	0.008	0.008	0.008	0.007	0.007
		Phoneme	0.001	0.001	0.001	0.001	0.001	0.001	0.001
		Pima Indians	0.005	0.005	0.005	0.004	0.006	0.005	0.005
		Skin	0.000	0.000	0.000	0.000	0.000	0.000	0.000
		SPECT	0.011	0.011	0.013	0.011	0.013	0.013	0.012
		Wilt	0.000	0.001	0.002	0.022	0.002	0.002	0.001
	50%	Haberman	0.007	0.007	0.008	0.008	0.008	0.007	0.007
		Phoneme	0.001	0.001	0.001	0.001	0.001	0.001	0.001
		Pima Indians	0.005	0.005	0.005	0.005	0.006	0.005	0.005
		Skin	0.000	0.000	0.000	0.000	0.000	0.000	0.000
		SPECT	0.011	0.011	0.013	0.012	0.013	0.014	0.012
		Wilt	0.000	0.001	0.001	0.001	0.002	0.001	0.000
RF	10%	Haberman	0.011	0.012	0.012	0.011	0.011	0.011	0.013
		Phoneme	0.002	0.002	0.002	0.002	0.002	0.002	0.002
		Pima Indians	0.006	0.007	0.007	0.007	0.006	0.006	0.007
		Skin	0.000	0.000	0.000	0.000	0.000	0.000	0.000
		SPECT	0.011	0.011	0.012	0.013	0.012	0.012	0.011
		Wilt	0.001	0.001	0.001	0.001	0.001	0.001	0.001
	30%	Haberman	0.012	0.013	0.013	0.012	0.014	0.014	0.013
		Phoneme	0.002	0.002	0.002	0.002	0.002	0.002	0.002
		Pima Indians	0.007	0.007	0.008	0.007	0.007	0.007	0.007
		Skin	0.000	0.000	0.000	0.000	0.000	0.000	0.000
		SPECT	0.013	0.011	0.011	0.011	0.011	0.011	0.012
		Wilt	0.001	0.001	0.001	0.001	0.001	0.001	0.001
	50%	Haberman	0.013	0.015	0.015	0.014	0.012	0.014	0.013
		Phoneme	0.002	0.002	0.002	0.002	0.002	0.002	0.002
		Pima Indians	0.008	0.007	0.007	0.007	0.007	0.008	0.007
		Skin	0.000	0.000	0.000	0.000	0.000	0.000	0.000
		SPECT	0.013	0.012	0.012	0.011	0.013	0.012	0.013
		Wilt	0.002	0.001	0.001	0.001	0.001	0.001	0.001
SVM	10%	Haberman	0.010	0.011	0.010	0.010	0.011	0.009	0.010
		Phoneme	0.003	0.003	0.002	0.003	0.003	0.003	0.002
		Pima Indians	0.006	0.006	0.006	0.007	0.006	0.006	0.006
		Skin	0.000	0.000	0.000	0.000	0.000	0.000	0.000
		SPECT	0.013	0.014	0.013	0.014	0.013	0.014	0.015
		Wilt	0.001	0.001	0.001	0.001	0.001	0.001	0.001
	30%	Haberman	0.010	0.011	0.010	0.012	0.011	0.012	0.010
		Phoneme	0.003	0.003	0.003	0.003	0.003	0.003	0.003
		Pima Indians	0.007	0.007	0.007	0.007	0.007	0.007	0.007
		Skin	0.000	0.000	0.000	0.000	0.000	0.000	0.000
		SPECT	0.013	0.013	0.014	0.014	0.015	0.013	0.017
		Wilt	0.001	0.001	0.001	0.001	0.001	0.001	0.001
	50%	Haberman	0.011	0.011	0.010	0.010	0.012	0.011	0.010
		Phoneme	0.003	0.003	0.003	0.003	0.003	0.003	0.003
		Pima Indians	0.008	0.008	0.006	0.009	0.009	0.006	0.007
		Skin	0.000	0.000	0.000	0.000	0.000	0.000	0.000
		SPECT	0.016	0.015	0.015	0.014	0.015	0.014	0.018
		Wilt	0.001	0.001	0.001	0.001	0.001	0.001	0.001
XGB	10%	Haberman	0.015	0.015	0.014	0.014	0.014	0.013	0.013
		Phoneme	0.002	0.002	0.002	0.002	0.002	0.002	0.002
		Pima Indians	0.008	0.009	0.008	0.009	0.008	0.010	0.010
		Skin	0.000	0.000	0.000	0.000	0.000	0.000	0.000
		SPECT	0.012	0.014	0.012	0.013	0.014	0.012	0.012
		Wilt	0.001	0.001	0.001	0.001	0.001	0.001	0.001
	30%	Haberman	0.014	0.015	0.014	0.014	0.015	0.015	0.015
		Phoneme	0.003	0.003	0.002	0.002	0.002	0.002	0.002
		Pima Indians	0.008	0.009	0.010	0.008	0.009	0.008	0.009
		Skin	0.000	0.000	0.000	0.000	0.000	0.000	0.000
		SPECT	0.014	0.013	0.012	0.013	0.013	0.012	0.012
		Wilt	0.001	0.001	0.001	0.001	0.001	0.001	0.001
	50%	Haberman	0.016	0.014	0.015	0.013	0.015	0.013	0.015
		Phoneme	0.003	0.002	0.002	0.003	0.002	0.003	0.003
		Pima Indians	0.008	0.008	0.009	0.009	0.010	0.009	0.008
		Skin	0.001	0.000	0.000	0.000	0.000	0.000	0.000
		SPECT	0.013	0.011	0.014	0.012	0.013	0.014	0.014
		Wilt	0.001	0.001	0.001	0.001	0.001	0.001	0.001

**Table A5:** Standard deviations of MMCE values for imputation methods under a MAR mechanism with *Two Thirds* pattern.

Classifier	% Miss.	Dataset	Hot Deck	MICE Norm	MICE PMM	MICE RF	missForest	missForest PMM	Mean
EN-LR	10%	Haberman	0.008	0.008	0.009	0.008	0.009	0.008	0.007
		Phoneme	0.001	0.001	0.001	0.001	0.001	0.001	0.001
		Pima Indians	0.005	0.005	0.005	0.005	0.005	0.005	0.005
		Skin	0.000	0.000	0.000	0.000	0.000	0.000	0.000
		SPECT	0.013	0.013	0.013	0.011	0.012	0.014	0.012
		Wilt	0.001	0.027	0.015	0.009	0.013	0.003	0.000
	30%	Haberman	0.007	0.008	0.007	0.009	0.009	0.007	0.009
		Phoneme	0.005	0.005	0.006	0.002	0.001	0.005	0.002
		Pima Indians	0.006	0.005	0.005	0.006	0.005	0.006	0.007
		Skin	0.002	0.000	0.001	0.001	0.001	0.003	0.000
		SPECT	0.015	0.015	0.015	0.014	0.014	0.014	0.015
		Wilt	0.000	0.001	0.016	0.009	0.001	0.002	0.000
	50%	Haberman	0.005	0.009	0.010	0.009	0.009	0.005	0.010
		Phoneme	0.001	0.000	0.002	0.006	0.005	0.002	0.007
		Pima Indians	0.009	0.007	0.007	0.006	0.006	0.008	0.006
		Skin	0.000	0.002	0.003	0.002	0.003	0.012	0.001
		SPECT	0.015	0.020	0.017	0.015	0.015	0.014	0.016
		Wilt	0.000	0.002	0.000	0.000	0.009	0.000	0.000
RF	10%	Haberman	0.012	0.011	0.013	0.012	0.013	0.013	0.012
		Phoneme	0.002	0.002	0.002	0.002	0.002	0.002	0.002
		Pima Indians	0.008	0.007	0.006	0.007	0.007	0.008	0.006
		Skin	0.000	0.000	0.000	0.000	0.000	0.000	0.000
		SPECT	0.012	0.012	0.012	0.012	0.011	0.014	0.012
		Wilt	0.001	0.001	0.001	0.001	0.001	0.001	0.001
	30%	Haberman	0.012	0.012	0.013	0.012	0.013	0.014	0.012
		Phoneme	0.003	0.003	0.003	0.003	0.003	0.003	0.003
		Pima Indians	0.008	0.008	0.007	0.007	0.008	0.009	0.007
		Skin	0.000	0.000	0.000	0.000	0.000	0.001	0.000
		SPECT	0.015	0.016	0.012	0.014	0.012	0.014	0.014
		Wilt	0.002	0.001	0.001	0.001	0.001	0.001	0.001
	50%	Haberman	0.014	0.012	0.015	0.014	0.014	0.015	0.014
		Phoneme	0.004	0.003	0.003	0.004	0.003	0.003	0.003
		Pima Indians	0.010	0.008	0.010	0.009	0.008	0.009	0.009
		Skin	0.001	0.001	0.000	0.000	0.000	0.001	0.000
		SPECT	0.016	0.016	0.014	0.014	0.015	0.015	0.014
		Wilt	0.001	0.001	0.001	0.001	0.001	0.002	0.001
SVM	10%	Haberman	0.009	0.011	0.009	0.009	0.010	0.010	0.009
		Phoneme	0.003	0.003	0.003	0.003	0.003	0.003	0.003
		Pima Indians	0.007	0.006	0.006	0.007	0.006	0.007	0.006
		Skin	0.000	0.000	0.000	0.000	0.000	0.000	0.000
		SPECT	0.012	0.013	0.014	0.014	0.013	0.014	0.014
		Wilt	0.001	0.001	0.001	0.001	0.001	0.001	0.001
	30%	Haberman	0.007	0.009	0.008	0.009	0.010	0.008	0.010
		Phoneme	0.004	0.004	0.003	0.004	0.003	0.004	0.003
		Pima Indians	0.009	0.007	0.007	0.008	0.008	0.008	0.008
		Skin	0.000	0.000	0.000	0.000	0.001	0.001	0.000
		SPECT	0.015	0.017	0.016	0.015	0.017	0.016	0.015
		Wilt	0.001	0.001	0.001	0.001	0.001	0.001	0.001
	50%	Haberman	0.007	0.008	0.008	0.009	0.010	0.006	0.012
		Phoneme	0.005	0.003	0.004	0.004	0.004	0.004	0.004
		Pima Indians	0.012	0.009	0.010	0.010	0.011	0.013	0.010
		Skin	0.001	0.000	0.000	0.000	0.001	0.003	0.000
		SPECT	0.015	0.016	0.018	0.015	0.014	0.016	0.012
		Wilt	0.002	0.001	0.001	0.001	0.001	0.002	0.001
XGB	10%	Haberman	0.012	0.015	0.013	0.013	0.013	0.014	0.014
		Phoneme	0.003	0.003	0.003	0.003	0.003	0.003	0.003
		Pima Indians	0.010	0.009	0.009	0.010	0.008	0.011	0.009
		Skin	0.000	0.000	0.000	0.000	0.000	0.000	0.000
		SPECT	0.015	0.014	0.012	0.014	0.013	0.014	0.012
		Wilt	0.001	0.001	0.001	0.001	0.001	0.001	0.001
	30%	Haberman	0.015	0.013	0.014	0.014	0.015	0.015	0.016
		Phoneme	0.004	0.003	0.003	0.003	0.003	0.004	0.004
		Pima Indians	0.010	0.010	0.011	0.010	0.008	0.012	0.009
		Skin	0.001	0.001	0.000	0.000	0.000	0.001	0.000
		SPECT	0.014	0.015	0.013	0.014	0.016	0.017	0.015
		Wilt	0.001	0.001	0.001	0.001	0.001	0.001	0.001
	50%	Haberman	0.015	0.013	0.014	0.014	0.016	0.016	0.013
		Phoneme	0.005	0.004	0.004	0.005	0.004	0.006	0.004
		Pima Indians	0.013	0.010	0.012	0.013	0.011	0.012	0.010
		Skin	0.002	0.001	0.001	0.001	0.001	0.002	0.001
		SPECT	0.016	0.016	0.014	0.015	0.014	0.016	0.014
		Wilt	0.002	0.001	0.001	0.001	0.002	0.002	0.001
